# Genomic Prediction and Genome-Wide Association Analysis of Egg Fertility and Hatchability Traits in Thai Native Grandparent Stock

**DOI:** 10.3390/ani16132004

**Published:** 2026-06-30

**Authors:** Veeraya Tantiyasawasdikul, Jiraporn Juiputta, Rawinan Lomngam, Vibuntita Chankitisakul, Wootichai Kenchaiwong, Wuttigrai Boonkum

**Affiliations:** 1 Department of Animal Science, Faculty of Agriculture, Khon Kean University, Khon Kean 40002, Thailand; veerayat@kkumail.com (V.T.); jiraporn.ju@kkumail.com (J.J.); lo_rawinan@kkumail.com (R.L.); vibuch@kku.ac.th (V.C.); 2 Network Center for Animal Breeding and Omics Research, Khon Kaen University, Khon Kaen 40002, Thailand; wootichai.k@msu.ac.th; 3 Small Ruminant Research Unit, Faculty of Veterinary Science, Mahasarakham University, Mahasarakham 44000, Thailand

**Keywords:** fertility, hatchability, genomic selection, genome-wide association study, accuracy

## Abstract

Fertility and hatchability are key reproductive traits affecting the efficiency and sustainability of poultry production; however, their genetic improvement remains challenging because of low heritability and complex biological control. In this study, we estimated the genetic parameters and compared the pedigree- and genomic-based prediction models for fertility rate (FER), hatchability of fertile eggs (HOF), and hatchability of eggs set (HOS) in Thai native chickens. In total, 7075 egg records from 1558 animals were analyzed, including pedigree data for 2646 individuals and genotypes for 400 animals. Prediction performance was evaluated using pedigree-based best linear unbiased prediction (PBLUP), single-step genomic BLUP (ssGBLUP), and weighted ssGBLUP (WssGBLUP). Additive heritability estimates for all traits ranged from low to moderate (0.051–0.068), indicating that environmental factors play an important role in the expression of these traits. Across all traits, WssGBLUP achieved the highest accuracy (0.647–0.648) and showed improved dispersion close to unity, indicating better model stability. Compared with PBLUP, WssGBLUP increased the prediction accuracy by 30–39%, respectively, whereas ssGBLUP outperformed PBLUP by approximately 14–22%. Genome-wide association analysis identified 65 candidate genes across multiple chromosomes, with a strong enrichment of significant signals on the Z chromosome, thus highlighting the role of sex-linked genetic variation. Individual loci explained small proportions of variance, confirming the polygenic nature of these traits. These findings demonstrate that weighted genomic approaches can substantially improve the accuracy and reliability of genetic evaluations for reproductive traits. Overall, this study provides practical support for implementing genomic selection in Thai native chickens, potentially contributing to enhanced reproductive performance, genetic progress, and sustainable poultry production.

## Simple Summary

Fertility and hatchability are important reproductive traits that directly influence chick production and the efficiency of poultry breeding programs. However, genetic improvement of these traits is challenging because they are influenced by many genes and environmental factors. This study evaluated fertility rate, hatchability of fertile eggs, and hatchability of eggs set in Thai native chickens using pedigree and genomic information. Three genetic evaluation methods were compared: pedigree-based BLUP (PBLUP), single-step genomic BLUP (ssGBLUP), and weighted single-step genomic BLUP (WssGBLUP). The results showed that genomic approaches improved prediction accuracy compared with pedigree-based evaluation, with WssGBLUP providing the highest accuracy and reliability. Several candidate genes associated with reproductive performance were identified, particularly on the Z chromosome. These findings support the implementation of genomic selection to improve reproductive efficiency and accelerate sustainable genetic improvement in Thai native chicken populations.

## 1. Introduction

Fertility and hatchability traits are key determinants of reproductive efficiency and chick output in poultry production systems [1,2,3]. These traits are particularly critical in native chickens because reproductive performance directly influences flock replacement, genetic dissemination, and the long-term sustainability of breeding programs [4,5,6]. Fertility rate (FER), hatchability of fertile eggs (HOF), and hatchability of eggs set (HOS) are biologically complex reproductive traits governed by multiple interrelated physiological processes, including gamete quality, fertilization efficiency, and early embryonic development. These processes are further influenced by genetic, environmental, and incubation-related factors [2,3,7]. Despite their economic importance, these traits typically exhibit low to moderate heritability, indicating substantial environmental influence and a highly polygenic genetic architecture [7,8,9]. Pedigree-based best linear unbiased prediction (PBLUP) has been widely applied for the genetic evaluation of reproductive traits in poultry breeding programs [7,10,11]. Although robust and computationally efficient, PBLUP relies on expected genetic relationships derived from pedigree records and therefore cannot fully capture Mendelian sampling variation among individuals within families [12]. These limitations are particularly pronounced for fertility traits, which are often sex-limited, expressed later in life, and recorded in only a subset of animals, thereby reducing the accuracy of estimated breeding values (EBVs) and hindering genetic progress [13]. Consequently, reliance on pedigree information alone may be insufficient to achieve substantial genetic gains for complex reproductive traits.

Genomic selection offers a powerful alternative by incorporating dense single-nucleotide polymorphism (SNP) information to capture the realized genetic relationships and improve the prediction accuracy for complex traits [14]. In poultry, genomic best linear unbiased prediction (GBLUP) and related approaches, including single-step GBLUP (ssGBLUP), have consistently demonstrated a superior predictive performance compared with that of pedigree-based models [15,16,17,18]. Nevertheless, there has been very little research on poultry reproductive traits using this method. Moreover, routinely applying GBLUP requires genotyping of all selection candidates, which may not be feasible in large-scale or resource-constrained breeding programs [19]. To address this limitation, ssGBLUP integrates pedigree, phenotypic, and genomic information into a unified relationship matrix, enabling simultaneous evaluation of genotyped and non-genotyped individuals [20,21]. This approach has improved prediction accuracy and reduced bias, particularly for low-heritability traits such as fertility and hatchability [14].

Weighted ssGBLUP (WssGBLUP) further extends ssGBLUP by iteratively assigning weights to SNP markers based on their estimated contribution to additive genetic variance, thereby accommodating heterogeneous genetic architectures [22,23]. This approach is particularly relevant for fertility traits that are expected to be influenced by a combination of polygenic background effects and genomic regions with moderate to large effects. By accounting for unequal SNP contributions, WssGBLUP can enhance prediction accuracy beyond standard ssGBLUP, particularly when trait architecture deviates from the infinitesimal model. In addition, genome-wide association studies in poultry have identified multiple genomic regions associated with reproductive performance, although most loci explain only a small proportion of the genetic variance, suggesting that fertility and hatchability are regulated by many genes of small effect. Consequently, genomic prediction approaches that utilize genome-wide marker information have become increasingly important for improving the accuracy of genetic evaluation and accelerating genetic gain for reproductive traits [24,25].

Thai native chickens (Pradu Hang Dum) have been developed through structured pure breeding and long-term selection to improve growth and egg production while maintaining adaptation to local production environments [26,27]. Despite their economic and genetic importance, genetic evaluations of fertility traits, particularly in eggs, in these populations have primarily relied on pedigree-based approaches, whereas comprehensive comparisons of advanced genomic evaluation methods remain limited [1,7]. Therefore, in this study, we aimed to (1) estimate the genetic parameters for fertility traits and (2) compare the accuracy and bias of EBVs obtained using PBLUP, ssGBLUP, and WssGBLUP in Thai native grandparent stock. We hypothesized that genomic-based methods would improve prediction accuracy compared with that of pedigree-based evaluation. By evaluating these approaches within a unified framework, this study provides insights into the optimal genomic evaluation strategy for improving fertility traits in native chicken breeding programs.

## 2. Materials and Methods

All experimental procedures were approved by the Institutional Animal Care and Use Committee (IACUC) of Khon Kaen University (KKU) under the Ethical Guidelines for Animal Experimentation of the National Research Council of Thailand (Approval No. IACUC-KKU-165/68).

### 2.1. Animals and Data Collection

The experiment was conducted at the experimental chicken farm of the Network Center for Animal Breeding and Omics Research, Faculty of Agriculture, KKU, Thailand. Records of egg fertility and hatchability traits, along with pedigree information, were collected from grandparent Thai native chickens (Pradu Hang Dum) during 2020–2026. In total, 7075 egg records for FER, HOF, and HOS were obtained from 1558 hens. Artificial insemination was performed twice weekly throughout the experimental period, and eggs were collected daily. The male-to-female ratio was maintained at 1:5. Hatching eggs were selected based on uniform size, normal shape, clean surface, and absence of shell defects. Eggs were stored for up to 7 days at 18–20 °C and 75% relative humidity before incubation. Incubation was carried out in electrically heated incubators under standard hatchery conditions. During the setter phase, air temperature and relative humidity were maintained at 37.5 °C and approximately 60%, respectively. The eggs were turned 24 times per day (once per hour) at an angle of approximately 45° per turn and candled on days 7 and 18 to remove infertile and dead (germ-dead) embryos. On day 18 of incubation, the eggs were transferred to a hatcher, where the temperature and RH were adjusted to 36.7–37.5 °C and approximately 70%, respectively. Traits were defined as follows:

$$\mathrm{FER}=\left(\frac{\mathrm{number}\;\mathrm{of}\;\mathrm{fertile}\;\mathrm{eggs}}{\mathrm{total}\;\mathrm{number}\;\mathrm{of}\;\mathrm{eggs}\;\mathrm{set}}\right)\times 100$$

$$\mathrm{HOF}=\left(\frac{\mathrm{number}\;\mathrm{of}\;\mathrm{chicks}\;\mathrm{hatched}}{\mathrm{number}\;\mathrm{of}\;\mathrm{fertile}\;\mathrm{eggs}}\right)\times 100$$

$$\mathrm{HOS}=\left(\frac{\mathrm{number}\;\mathrm{of}\;\mathrm{chicks}\;\mathrm{hatched}}{\mathrm{total}\;\mathrm{number}\;\mathrm{of}\;\mathrm{eggs}\;\mathrm{set}}\right)\times 100$$

### 2.2. Genomic DNA and SNP Profiles

Genomic DNA was collected from 400 descendant chickens, including equal numbers from maternal (n = 200) and paternal (n = 200) lineages. Genotyping was performed using the Axiom™ Genome-Wide Chicken 580K SNP Array mounted on a GeneTitan™ Multi-Channel platform (Thermo Fisher Scientific, Santa Clara, CA, USA). Quality control of genomic data was performed using the BLUPF90+ software package (version 3.06) [28]. Loci and individual samples were discarded if their genotyping call rates were lower than 90%. Additionally, Monomorphic SNPs and loci with minor allele frequencies (MAF) < 0.05 were removed. Genetic integrity across families was guaranteed through parentage-progeny audits; markers displaying a Mendelian mismatch rate exceeding 10% were removed, alongside individuals characterized by over 1% Mendelian discrepancies. This rigorous curation resulted in a final dataset containing 434,173 high-performing SNPs for downstream genomic modeling.

### 2.3. Statistical Analysis and Genetic Evaluation Models

Preliminary screening of the phenotypic data was performed using PROC UNIVARIATE in SAS version 9.0 to evaluate normality, homogeneity of variance, and the presence of outliers. Records exceeding three standard deviations from the mean were considered outliers and removed from the analysis. Bayesian multivariate repeatability models were fitted using the GIBBSF90 executable in the BLUPF90+ software package. The model was used to estimate additive heritability ($h_{a}^{2}$), mate sire heritability ($h_{ms}^{2}$), and permanent environmental variance ratio (pe^2^), and also derived phenotypic and genetic correlation statistics, along with individual estimated breeding values (EBVs). The model is defined as follows:

y=Xb+Za+Mms+Wpe+e

where **y** was the vector of observed phenotypes (FER, HOF, and HOS); **b** was the vector of fixed effects including the combination of hatch–month–year of insemination, generation, age of hen, and age of cock; **a** was the vector of random additive genetic effects; **ms** was the vector of random genetic effects of the mate sire; **pe** was the vector of random permanent environmental effects; **e** was the vector of random residual effects; and **X**, **Z**, **M**, and **W** were incidence matrices relating the observations to fixed, additive genetic, genetic effects of the mate sire, and permanent environmental effects, respectively. The variance–covariance structure used was as follows:

$$\mathrm{var}\left[\begin{array}{c} a\\ ms\\ pe\\ e \end{array}\right]=\left[\begin{array}{cccc} {A\sigma }_{a}^{2} & 0 & 0 & 0\\ 0 & {I\sigma }_{ms}^{2} & 0 & 0\\ 0 & 0 & {I\sigma }_{pe}^{2} & 0\\ 0 & 0 & 0 & {I\sigma }_{e}^{2} \end{array}\right]$$

where $\sigma_{a}^{2}$ was additive genetic variance, $\sigma_{ms}^{2}$ was genetic variance of the mate sire, $\sigma_{pe}^{2}$ was permanent environmental variance, $\sigma_{e}^{2}$ was residual variance, A was additive relationship matrix, and I was the identity matrix.

PBLUP, pedigree records were utilized to construct the numerator relationship matrix (**A**), which defined the genetic covariance structure among all individual birds. The additive genetic effects were postulated to follow a multivariate normal distribution characterized as:

$$a∼N\left(0,A\sigma_{a}^{2}\right)\;$$

where $\sigma_{a}^{2}$ represents the calculated additive genetic variance.

ssGBLUP, ssGBLUP was implemented to simultaneously analyze pedigree-based recorded and molecular profiles. In this design, the conventional inverse relationship structures within the operation mixed model equations are substituted with a synthesized relationship matrix $H^{-1}$. Following the structural formulation described by Legarra et al. [20] and Aguilar et al. [21], the generalized relationship array was developed as:

$$H^{-1}=A^{-1}+\left[\begin{array}{cc} 0 & 0\\ 0 & G^{-1}-A_{22}^{-1} \end{array}\right],$$

$H^{-1}$ was the integrated pedigree-genomic covariance matrix, $A^{-1}$ represented the inverted pedigree relationship matrix spanning all registered birds, $G^{-1}$ was the inverted genomic-derived relationship layer, and $A_{22}^{-1}$ denoted the traditional relationship values calculated exclusively for the genotyped subset.

WssGBLUP, to further capture the distinct, non-infinitesimal genetic footprints of the targeted traits, we applied WssGBLUP in a multi-trait repeatability setting. Unlike standard single-step models that treat marker contributions as uniform, WssGBLUP accounts for locus-specific variation by iteratively updating SNP weights according to their contribution to additive genetic variance [22]. Under this weighted genomic evaluation framework, the modified inverse of the combined relationship matrix (H^−1^) was incorporated into the mixed-model equations as follows:

$$H^{-1}=A^{-1}+\left[\begin{array}{cc} 0 & 0\\ 0 & \tau G^{-1}-\omega A_{22}^{-1} \end{array}\right],$$

where the scalar coefficients τ and *ω* represented scaling factors designed to adjust and balance the relative variances derived from genomic information (**G**) and those modeled through the pedigree subset (**A**_22_), thereby preventing potential inflation of prediction accuracy. The corresponding weighted relationship matrix (**G**) was structured using the strategy described by VanRaden [29]:

G=ZDZ′2∑i=1mpi(1−pi),di=1.125∣s^i∣SD(s^i)2,

within this system, **Z** denoted the genomic marker matrix containing gene content adjusted for ancestral allele frequencies, **D** represented a diagonal matrix acting as a weighting array for individual markers, and pi was the observed minor allele frequency at locus i. Weighted ssGBLUP was implemented using three consecutive weighting iterations. Previous studies reported that SNP weights and prediction accuracy generally converged after a limited number of iterations, typically between two and five rounds, with little additional improvement from further updates. Therefore, three iterations were considered sufficient to obtain stable SNP weight estimates while maintaining computational efficiency. SNP weights were updated using the nonlinear weighting function implemented in the BLUPF90+ software package, following the procedure described by Wang et al. [22]. The constants 1.125 and 2 were adopted from the original WssGBLUP framework and were used as scaling parameters to regulate the distribution of marker weights, thereby preventing excessive inflation of large-effect SNPs while maintaining the numerical stability of the genomic relationship matrix.

The diagonal elements of **D** (specifically d_i_) scale the variance contribution of each locus. In the initial iteration, all loci are treated equally (d_i_ = 1). For subsequent iterations, these weights are updated dynamically. The individual variance contribution of the ^th^SNP, denoted as ${\hat{s}}_{i}$, was the posterior mean SNP effect obtained from the threshold ssGBLUP analysis. All analyses were performed using a Gibbs sampling scheme with 120,000 computation cycles per analysis. To ensure convergence of the Markov chains, the first 30,000 iterations were discarded as burn-in. After this exclusion, a thinning interval was applied to retain only one out of every 20 sequential samples, thereby minimizing potential autocorrelation among consecutive sampling states. The statistical posterior means derived from these retained coordinates were used to estimate genetic variance, alongside traditional estimated breeding values (EBVs) and genomic estimated breeding values (GEBVs). Convergence was assessed using trace plots for the primary target parameters.

### 2.4. Model Comparison

To evaluate and compare the predictive capacity of the PBLUP, ssGBLUP, and WssGBLUP frameworks, we employed the linear regression (LR) method outlined by Legarra and Reverter [30]. This validation paradigm contrasts the breeding values (EBVs or GEBVs) estimated utilizing the complete historical dataset (w) against those generated from a truncated or partial dataset (p). The partial dataset was constructed by systematically masking the phenotypic observations. In this study, the partial dataset was created by randomly masking approximately 50% of the phenotypic records from animals in the validation population while retaining all pedigree and genomic information. This masking proportion was chosen to substantially reduce phenotypic information while preserving sufficient records for model training and validation. The approach follows the principles of the LR validation method, which assesses prediction bias, dispersion, and accuracy by comparing breeding values estimated from partial and complete datasets. The comparative performance and prediction quality of the models within this validation population were subsequently quantified using the following key statistics:

(1) Prediction Bias: To evaluate the systematic divergence between the breeding value estimates obtained from the restricted and complete databases, prediction bias was calculated as follows:

$$\mu_{w,p}={\bar{\hat{u}}}_{p}-{\bar{\hat{u}}}_{w},$$

where ${\bar{\hat{u}}}_{p}$ and ${\bar{\hat{u}}}_{w}$ represent the average estimated breeding values (including both pedigree and genomic predictions) computed from the partial and whole datasets, respectively.

(2) Prediction Accuracy: The reliability of prediction was characterized using the Pearson correlation coefficient between the breeding values generated from the restricted and complete computational runs. This metric acts as a proxy for the model’s capacity to consistently rank individual candidate birds, calculated as:

$$\rho_{w,p}=\frac{\mathrm{cov}({\hat{u}}_{p},{\hat{u}}_{w})}{\sqrt{\mathrm{var}({\hat{u}}_{p})\mathrm{var}({\hat{u}}_{w})}}$$

(3) Predictor Dispersion: To test for issues regarding inflation or deflation of the calculated evaluations, dispersion was determined by regressing the comprehensive estimate (${\hat{u}}_{w}$) onto the restricted counterparts (${\hat{u}}_{p}$). Under optimal conditions, a slope parameter equal to 1.00 signals the complete absence of under- or over-estimation, expressed as:

$$\beta_{w,p}=\frac{\mathrm{cov}({\hat{u}}_{w},{\hat{u}}_{p})}{\mathrm{var}({\hat{u}}_{p})}$$

(4) Covariance: The covariance structure linking the calculated breeding values between the partial and whole analytical designs was estimated to define their linear alignment, computed as:

$$\mathrm{cov}\left({\hat{u}}_{w},{\hat{u}}_{p}\right)$$

(5) Relative Percentage Gain in Accuracy (% Gain): The relative enhancement in predictive strength achieved by shifting from pedigree-guided systems (PBLUP accuracy; ${Acc}_{EBV}$) to single-step genomic structures (ssGBLUP and/or WssGBLUP accuracies; ${Acc}_{GEBV}$) was assessed. Comparisons were quantified using the proportional percentage change observed in the correlation-based accuracy estimates across the respective genomic and conventional models.

### 2.5. Genome-Wide Association Study (GWAS)

To identify genomic regions and specific nucleotide polymorphisms associated with fertility and hatchability performance, we conducted a GWAS using the optimal statistical model. SNP-specific marker effects were iteratively evaluated via the POSTGSF90 application interface. We back-calculated the estimated genomic breeding values ($\hat{u}$) to intermediate SNP effects ($\hat{s}$) partitionable by the overall genomic variance ($\sigma_{u}^{2}$), applying the following matrix notation:

$$\hat{s}=DZ^{\prime }G^{-1}\hat{u},$$

where D represents a matrix of SNP weights, Z denotes the incidence matrix relating SNP genotypes to individuals, and G represents the constructed genomic relationship matrix. The percentage of total additive genetic variation accounted for by local chromosomal regions—defined here as five adjacent SNPs—was quantified as follows:

$$\frac{Var\left(\sum_{j=1}^{5}z_{j}{\hat{s}}_{j}\right)}{{\hat{\sigma }}_{u}^{2}}\times 100,$$

where $Var\left(\sum_{j=1}^{5}z_{j}{\hat{s}}_{j}\right)$ is the localized genetic variance attributable to a specific 5-SNP window, ${\hat{\sigma }}_{u}^{2}$ represents the total additive genetic variance, $z_{j}$ acts as the vector of allele dosage for the $j^{th}$ marker, and ${\hat{s}}_{j}$ denotes the corresponding marker effect evaluation. Additionally, we measured the proportion of genetic variance captured across the chicken genome using a 1-Mb sliding window approach. Genomic segments accounting ≥ 0.5% of the total additive genetic variance were prioritized as candidate regions of interest for reproduction traits. To locate positional candidate genes within these identified windows, we cross-referenced their physical coordinates with the *Gallus gallus* reference genome assembly (GRCg6a). Genetic annotation was run using the R biomaRt package (version 2.66.0) connected to the Ensembl Release 99 archive (dated January 2020) to maintain structural alignment with the assembly coordinates. We compiled all protein-coding genes mapped within the significant 1-Mb windows for downstream curation. Functional enrichment analysis was performed using the DAVID Bioinformatics platform (Knowledgebase version v. 2025_2) to discover overrepresented biological pathways and molecular characteristics. Manhattan plots were generated in R (RStudio version 2025.09.1) to visualize SNP-associated genetic variance across the genome.

## 3. Results

### 3.1. Egg Fertility Performance

The descriptive statistics of the dataset used for variance component estimation are presented in Table 1. A total of 7075 egg records were obtained from 1558 animals with phenotypic records, whereas 2646 animals were included in the pedigree. Genotype information was available for 400 individuals, and the dataset included 609 mating sires. The average age at first egg was 180 ± 26 days. Egg weight increased with age, averaging 37.93 ± 2.28 g at first egg, 46.63 ± 2.45 g at 240 days of age, and 52.98 ± 3.93 g at 365 days of age. Fertility and hatchability traits varied among hatches. The mean fertility rate (FER) was 88.85 ± 30.23%, 89.16 ± 32.28%, and 82.50 ± 33.26% for hatches 1, 2, and 3, respectively. Hatchability of fertile eggs (HOF) averaged 87.39 ± 30.08%, 85.69 ± 30.14%, and 77.92 ± 26.12% across hatches 1, 2, and 3, respectively. Similarly, hatchability of eggs set (HOS) averaged 78.07 ± 28.05%, 77.56 ± 28.10%, and 74.96 ± 24.09% for hatches 1, 2, and 3, respectively.

### 3.2. Variance Components and Genetic Parameter Estimates

The variance components and genetic parameter estimates for FER, HOF, and HOS were obtained using the PBLUP, ssGBLUP, and WssGBLUP models and are presented in Table 2. Additive genetic variance ($\sigma_{a}^{2}$) was generally consistent across models, although slightly higher estimates were observed for the genomic-based approaches. For FER, $\sigma_{a}^{2}$ increased from 40.55 under PBLUP to 42.77 and 43.59 under ssGBLUP and WssGBLUP, respectively. Similar increases were observed for HOF (64.91–68.95) and HOS (50.81–55.45). The mate sire genetic variance ($\sigma_{ms}^{2}$) remained relatively stable across models for all traits, ranging from 91.06 to 91.40 for FER, 118.48 to 120.16 for HOF, and 78.99 to 79.43 for HOS. Permanent environmental variance ($\sigma_{pe}^{2}$) and residual variance ($\sigma_{e}^{2}$) showed only minor differences among models. For FER, $\sigma_{pe}^{2}$ ranged from 76.99 to 78.59, whereas $\sigma_{e}^{2}$ ranged from 426.31 to 430.86. For HOF, $\sigma_{pe}^{2}$ varied between 155.61 and 177.27, while $\sigma_{e}^{2}$ ranged from 895.69 to 898.41. Similarly, for HOS, $\sigma_{pe}^{2}$ ranged from 177.21 to 213.12, and $\sigma_{e}^{2}$ ranged from 667.79 to 669.29.

Estimates of additive heritability ($h_{a}^{2}$) were low across all traits and models. For FER, $h_{a}^{2}$ ranged from 0.063 ± 0.02 under PBLUP to 0.068 ± 0.02 under WssGBLUP. For HOF, estimates ranged from 0.052 ± 0.01 to 0.055 ± 0.01, whereas HOS showed values ranging from 0.051 ± 0.01 to 0.057 ± 0.01. Mate sire heritability ($h_{ms}^{2}$) was consistently higher than additive heritability across all traits. For FER, $h_{ms}^{2}$ ranged from 0.142 ± 0.06 to 0.143 ± 0.05. Corresponding estimates for HOF and HOS ranged from 0.094 ± 0.03 to 0.097 ± 0.04 and from 0.078 ± 0.03 to 0.081 ± 0.03, respectively. The permanent environmental variance ratio (pe^2^) varied among traits and models. For FER, pe^2^ ranged from 0.121 ± 0.05 to 0.123 ± 0.05. For HOF, values ranged from 0.126 ± 0.05 to 0.141 ± 0.05. Higher estimates were observed for HOS, ranging from 0.181 ± 0.07 to 0.210 ± 0.08.

### 3.3. Genetic and Phenotypic Correlation Estimates

The genetic (above diagonal) and phenotypic (below diagonal) correlations among FER, HOF, and HOS were estimated using PBLUP, ssGBLUP, and WssGBLUP, and were presented in Table 3. Under the PBLUP model, genetic correlations among traits were high and positive, ranging from 0.799 to 0.878. The strongest correlation was observed between HOF and HOS (0.878), followed by FER–HOF (0.845) and FER–HOS (0.799). Corresponding phenotypic correlations were moderate to high, with estimates of 0.625, 0.583, and 0.854 for FER–HOF, FER–HOS, and HOF–HOS, respectively. Under the ssGBLUP model, genetic correlations followed a similar pattern but were slightly higher, ranging from 0.821 to 0.889. The highest estimate was again observed between HOF and HOS (0.889), followed by FER–HOF (0.864) and FER–HOS (0.821). Phenotypic correlations were 0.689, 0.630, and 0.867 for FER–HOF, FER–HOS, and HOF–HOS, respectively. Estimates obtained from the WssGBLUP model were consistently higher than those from the other approaches. Genetic correlations ranged from 0.835 to 0.897, with the strongest association again observed between HOF and HOS (0.897), followed by FER–HOF (0.872) and FER–HOS (0.835). Phenotypic correlations were 0.699, 0.638, and 0.872 for FER–HOF, FER–HOS, and HOF–HOS, respectively.

### 3.4. Genomic Prediction Model Performance and Stability

The performance and stability of genomic prediction models for FER, HOF, and HOS based on the LR validation method were compared and presented in Table 4. For FER, the lowest bias estimate was observed for WssGBLUP (0.015), followed by ssGBLUP (0.020) and PBLUP (0.060). Dispersion estimates differed among models, with WssGBLUP (1.083) being closest to unity, whereas ssGBLUP (0.337) and PBLUP (0.217) deviated substantially from unity. WssGBLUP achieved the highest prediction accuracy (0.648), followed by ssGBLUP (0.533) and PBLUP (0.466). Covariance estimates were also greater for WssGBLUP (0.239) than for PBLUP (0.066) and ssGBLUP (0.031). Relative to PBLUP and ssGBLUP, WssGBLUP improved prediction accuracy by 39.07% and 21.64%, respectively, whereas ssGBLUP improved prediction accuracy by 12.53% compared with PBLUP.

For HOF, bias estimates were 0.046, 0.080, and 0.062 for PBLUP, ssGBLUP, and WssGBLUP, respectively. Dispersion estimates were 0.895, 0.731, and 1.391 for PBLUP, ssGBLUP, and WssGBLUP, respectively. WssGBLUP again produced the highest prediction accuracy (0.646), followed by ssGBLUP (0.562) and PBLUP (0.491). Covariance estimates were also highest for WssGBLUP (5.869), compared with 3.998 for PBLUP and 3.202 for ssGBLUP. The WssGBLUP model increased prediction accuracy by 31.55% relative to PBLUP and by 14.93% relative to ssGBLUP, whereas ssGBLUP improved prediction accuracy by 12.64% compared with PBLUP.

For HOS, bias estimates were 0.077 for PBLUP, 0.053 for ssGBLUP, and 0.036 for WssGBLUP. Dispersion estimates were 0.774, 0.840, and 1.562 for PBLUP, ssGBLUP, and WssGBLUP, respectively. WssGBLUP achieved the highest prediction accuracy (0.647), followed by ssGBLUP (0.566) and PBLUP (0.497). Covariance estimates were 3.239, 1.997, and 4.734 for PBLUP, ssGBLUP, and WssGBLUP, respectively. Compared with PBLUP and ssGBLUP, WssGBLUP increased prediction accuracy by 30.28% and 14.47%, respectively, whereas ssGBLUP improved prediction accuracy by 12.14% relative to PBLUP.

### 3.5. Genome-Wide Association Analysis

Genome-wide association analysis identified 65 candidate genes associated with FER, HOF, and HOS was presented in Table 5. Significant SNPs were distributed across multiple chromosomes, including chromosomes Z, 1, 2, 3, 4, 5, 7, 8, and 17. A higher concentration of significant SNPs was observed on chromosome Z, particularly within the region spanning 79.1–81.9 Mb. Additional clusters of associated SNPs were identified on chromosome 5 (28.3–29.1 Mb) and chromosome 2 (38.1–39.0 Mb). The proportion of phenotypic variance explained by individual SNPs ranged from 0.006 to 0.010, with the largest effects detected for SNPs located on chromosome Z. A total of 65 candidate genes were identified, including *PLEKHH1*, *DCAF5*, *EXD2*, *VCAN*, *XRCC4*, *RAD51B*, *ZMYND11*, *KDM6A*, *EOMES*, *ARG2*, *SLC4A7*, *GNPDA2*, *YIPF7*, *RBMS3*, and *NHLH2*. These genes were located within multiple genomic regions associated with the reproductive traits evaluated in this study. Most candidate genes were associated with all three traits (FER, HOF, and HOS). In contrast, a subset of genes, including *EFHC1*, *IL17F*, *MCM3*, *PAQR8*, and *TRAM2*, showed trait-specific associations with HOF and HOS.

## 4. Discussion

The present study provided a comprehensive evaluation of egg fertility and hatchability traits in Thai native grandparent chickens by integrating phenotypic, pedigree, and genomic information. The results highlighted the biological complexity of reproductive traits and demonstrated the potential of genomic approaches to improve prediction accuracy in poultry breeding programs. Fertility rate (FER), hatchability of fertile eggs (HOF), and hatchability of eggs set (HOS) showed moderate variation across hatches, reflecting the multifactorial nature of these traits. In poultry, reproductive performance is influenced by numerous factors, including gamete quality, fertilization efficiency, embryonic development, and environmental conditions, which collectively contribute to the generally low to moderate heritability of these traits [1,8,31].

The descriptive statistics revealed relatively high mean values for FER, HOF, and HOS, accompanied by considerable variation across hatches. This finding was consistent with previous reports in poultry populations, in which reproductive traits exhibited substantial phenotypic variability owing to environmental factors such as nutrition, management, and incubation conditions [32,33]. The decline in fertility and hatchability observed across successive hatches may have reflected changes in the physiological status of hens, sperm storage efficiency, or environmental stressors affecting reproductive performance. These findings highlighted the importance of accounting for both fixed and random effects in genetic evaluation models.

The estimated additive heritability for fertility and hatchability traits in this study (0.051–0.068) fell within the range commonly reported for reproductive traits in poultry, which are generally characterized by low heritability [7,8,34,35]. This finding suggested that environmental factors and non-additive genetic effects played substantial roles in the expression of these traits. The low heritability estimates further indicated that traditional selection methods based solely on phenotypic or pedigree information were less effective, thereby highlighting the importance of genomic approaches that captured additional sources of genetic variation. An interesting finding of the present study was that mate sire heritability estimates were consistently higher than direct additive heritability estimates across all fertility and hatchability traits. This result suggested that paternal genetic effects contributed substantially to reproductive performance in Thai native chickens. The mate sire component likely reflected genetic variation associated with sperm production, motility, viability, storage efficiency within the hen reproductive tract, fertilization capacity, and paternal influences on early embryonic development [36,37,38]. Previous studies reported that sperm DNA integrity, chromatin stability, and genome maintenance mechanisms were important determinants of fertility and embryo survival in poultry [39,40]. Therefore, the relatively higher mate sire heritability observed in this study may have reflected the combined influence of these paternal biological processes.

Comparison of prediction models using the LR validation method revealed clear differences in predictive performance among PBLUP, ssGBLUP, and WssGBLUP. Across all traits, WssGBLUP consistently achieved the highest prediction accuracy, followed by ssGBLUP and PBLUP. These findings were consistent with previous studies that demonstrated that genomic-based models, particularly ssGBLUP and WssGBLUP, outperformed pedigree-based BLUP approaches in poultry genetic evaluations [14,17,41,42]. The superior performance of WssGBLUP was attributed to its ability to assign differential weights to SNP effects, thereby providing a more accurate representation of trait-specific genetic architecture and improving prediction accuracy compared with standard GBLUP and ssGBLUP models [43,44,45].

Bias estimates across models were generally low, indicating minimal systematic over- or underestimation of breeding values. However, dispersion coefficients revealed notable differences in model stability. WssGBLUP produced dispersion coefficients closest to unity for FER, suggesting more appropriate scaling of predicted breeding values than PBLUP and ssGBLUP. Dispersion coefficients substantially below 1, as observed for PBLUP and ssGBLUP in some traits, indicated inflation or deflation of predictions, which may have reduced the effectiveness of selection decisions [30]. The improved dispersion observed for WssGBLUP further supported its suitability for the genetic evaluation of complex reproductive traits. The improvement in prediction accuracy achieved using WssGBLUP relative to PBLUP (30–39%) and ssGBLUP (14–22%) was substantial. These results highlighted the practical value of incorporating genomic information into breeding programs, particularly for traits that were difficult to measure or exhibited low heritability. In Thai native chickens, where reproductive performance was critical for sustainable production systems, such gains could potentially translate into substantial improvements in productivity and profitability.

Functionally, the candidate genes identified in the present study were classified into three categories based on the strength of biological evidence: (1) genes with strong evidence for reproductive or developmental functions, (2) genes with moderate evidence through indirect physiological pathways, and (3) positional candidate genes with limited direct evidence linking them to reproductive performance. Among the most promising candidates, *XRCC4*, *RAD51B*, and *EXD2* were involved in DNA repair and genome maintenance processes that are essential for meiosis, gametogenesis, and early embryonic development, in which genomic integrity plays a critical role in embryo survival and reproductive success [46,47,48,49]. Similarly, *KDM6A*, *ZMYND11*, and *EOMES* were associated with epigenetic regulation, transcriptional control, and developmental processes that may influence embryonic competence and reproductive performance [50,51,52,53]. Given their documented roles in cellular processes directly related to reproduction and development, these genes were considered the most biologically plausible candidates underlying variation in fertility and hatchability traits.

A second group of genes contributed indirectly to reproductive performance through the regulation of physiological pathways that influenced cellular homeostasis and metabolic status. For example, *SLC4A7* participated in bicarbonate and ion transport [54], whereas *ARG2* and *GNPDA2* were involved in amino acid metabolism, energy balance, and metabolic regulation [55,56,57]. These pathways may have influenced reproductive efficiency by affecting the cellular environment required for fertilization, embryonic development, and the maintenance of reproductive tissues. Although direct evidence linking these genes to fertility traits in chickens was limited, their known physiological functions provided a plausible biological basis for further investigation.

The third group comprised positional candidate genes, including *YIPF7*, *RFWD2*, and *FNBP1*, for which direct evidence linking them to avian fertility or hatchability was limited. These genes were identified because they were located within genomic regions that explained a proportion of the additive genetic variance; however, they could not be considered causal genes based solely on the present GWAS results. Their potential involvement in reproductive traits may have arisen through indirect effects on cellular organization, protein turnover, intracellular trafficking, or other biological processes that remain poorly characterized in poultry. Therefore, these genes should be interpreted cautiously as positional candidates requiring further functional validation. The identification of several candidate genes shared across FER, HOF, and HOS suggested the presence of common biological mechanisms underlying fertilization and embryonic development. In contrast, the detection of trait-specific genomic regions indicated that these reproductive traits were not entirely controlled by the same genetic factors, which is consistent with previous reports describing both shared and distinct genetic architectures among fertility-related traits [31,58].

From a breeding perspective, the identified genomic regions provided useful information for genomic prediction and the refinement of genomic evaluation models. However, the relatively small proportion of genetic variance explained by individual loci and the large number of associated regions detected across the genome supported the conclusion that fertility and hatchability were highly polygenic traits controlled by numerous loci with small effects. Therefore, these findings were likely to be more valuable for genomic selection than for marker-assisted selection based on individual loci. Furthermore, the concentration of associated signals on the Z chromosome highlighted the potential importance of sex-linked genetic variation and supported the inclusion of sex-chromosome information in future genomic evaluation and breeding strategies. Overall, the candidate genes identified in this study should be regarded as biologically informative hypotheses rather than definitive causal determinants of reproductive performance. Further validation through fine-mapping, gene expression studies, and functional analyses will be necessary to confirm their roles in fertility and hatchability traits.

From a biological perspective, the complexity of fertility and hatchability traits was further compounded by their dependence on both male and female reproductive functions. In poultry, fertility was determined by semen quality, sperm survival and storage within the hen’s reproductive tract, and successful fertilization, whereas hatchability was primarily influenced by embryonic development, embryo survival, and incubation conditions [59,60]. Oxidative stress, nutrient availability, and environmental factors such as temperature also affected these processes, contributing to variation in reproductive performance [61,62]. Therefore, the integration of genomic, physiological, and environmental information represented a promising strategy for improving genetic prediction and selection. In the present study, we characterized the genomic architecture underlying fertility and hatchability and identified loci distributed across multiple chromosomes, further supporting the polygenic nature of these traits. The broad distribution of significant SNPs and their relatively small individual effects were consistent with previous GWAS studies of reproductive traits in livestock, which have generally been shown to be influenced by numerous loci with small effects [63,64].

However, the absence of validation in independent populations limited the robustness of these findings, and functional characterization of the identified candidate genes remained necessary. In the present study, genomic windows explaining ≥0.5% of the total additive genetic variance were prioritized as candidate regions according to the WssGWAS framework, which was used as an exploratory approach to identify genomic regions contributing relatively greater proportions of genetic variation. Because this variance-based approach did not rely on formal single-marker hypothesis testing, these regions should not be interpreted as genome-wide significant loci under conventional Bonferroni- or false discovery rate (FDR)-adjusted thresholds. Therefore, the genes located within these regions should be considered putative candidate genes whose biological relevance requires validation in larger, independent populations. Furthermore, integrating GWAS results with transcriptomic and functional genomic analyses will be essential for identifying causal variants and strengthening the biological interpretation of these associations.

Several limitations of the present study should be considered when interpreting the results. First, fertility rate (FER), hatchability of fertile eggs (HOF), and hatchability of eggs set (HOS) were proportional traits bounded between 0 and 100%. Although preliminary diagnostic analyses indicated that their distributions were sufficiently close to normal to justify the use of linear mixed models, generalized linear mixed models (GLMMs) with appropriate link functions may provide a more flexible framework by explicitly accounting for the binomial nature of the data and heterogeneous variance structures. Nevertheless, linear mixed models remained widely used in animal breeding and genomic evaluation because they were computationally efficient and readily integrated with PBLUP, ssGBLUP, and WssGBLUP evaluation systems. Future studies should compare linear and generalized linear genomic evaluation approaches to determine whether additional improvements in parameter estimation and predictive accuracy can be achieved for fertility and hatchability traits.

In chickens, females are heterogametic (ZW) and carry only one copy of the Z chromosome, whereas males are homogametic (ZZ). The genomic relationship matrix used in the present study was constructed using the standard VanRaden formulation, which was primarily developed for diploid autosomal markers and did not explicitly account for the hemizygous state of Z-linked loci in females. Therefore, SNP effect estimates and local genomic variance estimates on the Z chromosome may have been influenced by sex-specific differences in allele dosage. Because several candidate regions were identified on the Z chromosome, these associations should be interpreted cautiously as putative Z-linked signals. Future studies should incorporate sex chromosome-specific genomic relationship matrices or sex-specific marker coding to better account for hemizygosity, dosage compensation, and potential sex-specific genetic effects.

Finally, the number of genotyped animals (n = 400) was relatively small compared with the high density of SNP markers analyzed. Although this sample size was generally sufficient for single-step genomic evaluation, it may have limited the statistical power of the GWAS to detect loci with small effects and increased the likelihood of false-positive associations. Because fertility and hatchability are complex polygenic traits with generally low heritability, larger reference populations are typically required for the reliable identification of causal variants. Nevertheless, the integration of genomic, pedigree, and phenotypic information through ssGBLUP and WssGBLUP enabled efficient use of the available data and substantially improved prediction accuracy compared with pedigree-based evaluation. Therefore, the genomic regions identified in this study should be considered putative candidate loci that require validation in larger populations and independent Thai native chicken lines before being incorporated into marker-assisted or genomic selection programs.

## 5. Conclusions

This study demonstrated that egg fertility and hatchability traits in Thai native chickens were complex traits with low to moderate heritability, which may have limited the effectiveness of pedigree-based selection. Genomic prediction models improved predictive performance, with WssGBLUP providing greater accuracy and stability than PBLUP and ssGBLUP. Genome-wide association analysis identified 65 candidate genes, and significant SNPs were distributed across multiple chromosomes, with a notable enrichment on the Z chromosome, supporting the polygenic nature of these traits. Overall, the findings supported the application of weighted genomic approaches for improving the genetic evaluation and selection of reproductive traits in Thai native chickens. Future studies should focus on expanding the reference population and integrating functional genomic and environmental information to further improve prediction accuracy.

## Figures and Tables

**Table 1:** Summary of the data used for variance component estimations, with values presented as mean ± standard deviation.

Category	N	FER	HOF	HOS
Number of egg records (n)	7075	-	-	-
Animals with records (n)	1558	-	-	-
Animals with pedigree records (n)	2646	-	-	-
Animals with genotypes (n)	400	-	-	-
Number of mate sires (n)	609	-	-	-
Average age of hens at first egg (day)	180 ± 26	-	-	-
Average egg weight (g) - At first egg - At 240 days of age - At 365 days of age	37.93 ± 2.28 46.63 ± 2.45 52.98 ± 3.93	- - -	- - -	- - -
Egg fertility traits by hatch (%)	1 2 3	88.85 ± 30.23 89.16 ± 32.28 82.50 ± 33.26	87.39 ± 30.08 85.69 ± 30.14 77.92 ± 26.12	78.07 ± 28.05 77.56 ± 28.10 74.96 ± 24.09

FER = fertility rate, HOF = hatchability of fertile eggs, HOS = hatchability of eggs set.

**Table 2:** Comparison of variance components and genetic parameter estimates for egg fertility and hatchability traits (FER, HOF, and HOS) under PBLUP, ssGBLUP, and WssGBLUP models.

Parameters	Method								
	PBLUP			ssGBLUP			WssGBLUP		
	FER	HOF	HOS	FER	HOF	HOS	FER	HOF	HOS
$\sigma_{a}^{2}$	40.55	64.91	50.81	42.77	67.37	55.28	43.59	68.95	55.45
$\sigma_{ms}^{2}$	91.16	120.16	78.99	91.40	119.65	79.43	91.06	118.48	79.24
$\sigma_{pe}^{2}$	78.59	155.61	206.34	78.11	160.77	213.12	76.99	177.27	177.21
$\sigma_{e}^{2}$	430.86	898.41	669.29	428.73	896.20	668.41	426.31	895.69	667.79
$h_{a}^{2}$ ± SE	0.063 ±0.02	0.052 ±0.01	0.051 ±0.01	0.067 ±0.02	0.054 ±0.01	0.054 ±0.01	0.068 ±0.02	0.055 ±0.01	0.057 ±0.01
$h_{ms}^{2}$ ± SE	0.142 ±0.06	0.097 ±0.04	0.079 ±0.03	0.143 ±0.05	0.096 ±0.03	0.078 ±0.03	0.143 ±0.05	0.094 ±0.03	0.081 ±0.03
${pe}^{2}$ ± SE	0.123 ±0.05	0.126 ±0.05	0.205 ±0.08	0.122 ±0.05	0.129 ±0.05	0.210 ±0.08	0.121 ±0.05	0.141 ±0.05	0.181 ±0.07

$\sigma_{a}^{2}$ represents additive genetic variance, $\sigma_{ms}^{2}$ represents genetic variance of mate sire, $\sigma_{pe}^{2}$ represents permanent environmental variance, $\sigma_{e}^{2}$ represents residual variance, $h_{a}^{2}$ represents additive heritability, $h_{ms}^{2}$ represents mate sire heritability, ${pe}^{2}$ represents permanent environmental variance ratio. FER = fertility rate, HOF = hatchability of fertile eggs, HOS = hatchability of eggs set, PBLUP = pedigree-based best linear unbiased prediction, ssGBLUP = single-step genomic best linear unbiased prediction, WssGBLUP = weighted single-step genomic best linear unbiased prediction.

**Table 3:** Genetic (above diagonal) and phenotypic (below diagonal) correlations among egg fertility and hatchability traits using PBLUP, ssGBLUP, and WssGBLUP models.

**PBLUP/Traits**	**FER**	**HOF**	**HOS**
FER	-	0.845	0.799
HOF	0.625	-	0.878
HOS	0.583	0.854	-
**ssGBLUP/Traits**	**FER**	**HOF**	**HOS**
FER	-	0.864	0.821
HOF	0.689	-	0.889
HOS	0.630	0.867	-
**WssGBLUP/Traits**	**FER**	**HOF**	**HOS**
FER	-	0.872	0.835
HOF	0.699	-	0.897
HOS	0.638	0.872	-

FER = fertility rate, HOF = hatchability of fertile eggs, HOS = hatchability of eggs set, PBLUP = pedigree-based best linear unbiased prediction, ssGBLUP = single-step genomic best linear unbiased prediction, WssGBLUP = weighted single-step genomic best linear unbiased prediction.

**Table 4:** Comparison of the genomic prediction model performance and stability for egg fertility and hatchability traits using the Linear Regression (LR) method.

Traits	Statistic Criteria	PBLUP	ssGBLUP	WssGBLUP
FER	Bias	0.060	0.015	0.020
	Dispersion	0.217	0.337	1.083
	Accuracy	0.466	0.533	0.648
	Covariance	0.066	0.031	0.239
	% Gain (ssGBLUP vs. PBLUP)	-	12.530	-
	% Gain (WssGBLUP vs. ssGBLUP)	-	-	21.640
	% Gain (WssGBLUP vs. PBLUP)	-	-	39.070
HOF	Bias	0.046	0.080	0.062
	Dispersion	0.895	0.731	1.391
	Accuracy	0.491	0.562	0.646
	Covariance	3.998	3.202	5.869
	% Gain (ssGBLUP vs. PBLUP)	-	12.640	-
	% Gain (WssGBLUP vs. ssGBLUP)	-	-	14.930
	% Gain (WssGBLUP vs. PBLUP)	-	-	31.550
HOS	Bias	0.077	0.053	0.036
	Dispersion	0.774	0.840	1.562
	Accuracy	0.497	0.566	0.647
	Covariance	3.239	1.997	4.734
	% Gain (ssGBLUP vs. PBLUP)	-	12.140	-
	% Gain (WssGBLUP vs. ssGBLUP)	-	-	14.470
	% Gain (WssGBLUP vs. PBLUP)	-	-	30.280

FER = fertility rate, HOF = hatchability of fertile eggs, HOS = hatchability of eggs set, PBLUP = pedigree-based best linear unbiased prediction, ssGBLUP = single-step genomic best linear unbiased prediction, WssGBLUP = weighted single-step genomic best linear unbiased prediction.

**Table 5:** Candidate genes associated with egg fertility and hatchability traits identified through genome-wide association study (GWAS).

NO.	SNP ID	Variance	Chromosome	Location (bp)	Candidate Genes	Gene Size (bp)	Distance (bp)	Putative Function	Associated Traits
1	430341	0.010	Z	80,206,648	*LOC112530552*	95,699	−18,111	-	FER, HOF, HOS
2	430380	0.010	Z	80,386,260	*LOC112530555*	1236	−26,265	-	FER, HOF, HOS
3	430436	0.010	Z	80,714,030	*LOC112530563*	56,937	−6873	-	FER, HOF, HOS
4	430436	0.010	Z	80,714,030	*LOC112530568*	2742	−34,722	-	FER, HOF, HOS
5	430406	0.010	Z	80,504,842	*LOC112530557*	801	13,035	-	FER, HOF, HOS
6	430406	0.010	Z	80,504,842	*LOC112530558*	1582	−30,184	-	FER, HOF, HOS
7	430434	0.010	Z	80,684,089	*LOC112530559*	1372	−1476	-	FER, HOF, HOS
8	430228	0.009	Z	79,540,061	*LOC112530635*	18,868	−11,865	-	FER, HOF, HOS
9	430191	0.008	Z	79,322,762	*LOC112530770*	1763	−6115	-	FER, HOF, HOS
10	218967	0.008	5	29,039,532	*LOC101749472*	6988	46,159	-	FER, HOF, HOS
11	218967	0.008	5	29,039,532	*LOC107053490*	11,871	16,838	-	FER, HOF, HOS
12	218967	0.008	5	29,039,532	*LOC112532518*	2325	27,794	-	FER, HOF, HOS
13	218967	0.008	5	29,039,532	*PLEKHH1*	55,381	29,111	Cytoskeleton-associated protein with FERM, MyTH4, and PH domains, potentially involved in membrane–cytoskeleton interaction and cellular structural organization.	FER, HOF, HOS
14	218601	0.007	5	28,325,310	*DCAF5*	66,506	−14,304	Protein-binding component involved in ubiquitin-mediated protein modification and regulation of fatty acid biosynthesis.	FER, HOF, HOS
15	218601	0.007	5	28,325,310	*EXD2*	21,681	2626	Encodes a 3′-to-5′ exonuclease/nuclease involved in nucleic acid binding, DNA double-strand break processing, homologous recombination repair, and replication fork processing.	FER, HOF, HOS
16	428437	0.007	Z	62,962,710	*MIR1756A*	90	−33,522	-	FER, HOF, HOS
17	428437	0.007	Z	62,962,710	*VCAN*	106,376	107,986	Secreted extracellular matrix glycoprotein/proteoglycan involved in cell adhesion, hyaluronic acid binding, carbohydrate binding, and tissue development.	FER, HOF, HOS
18	428437	0.007	Z	62,962,710	*XRCC4*	180,792	−29,954	XRCC4-like DNA repair protein involved in non-homologous end joining, DNA double-strand break repair, DNA ligase IV complex function, and genome stability.	FER, HOF, HOS
19	218987	0.007	5	29,090,184	*RDH12*	6972	−9883	-	FER, HOF, HOS
20	218987	0.007	5	29,090,184	*VTI1B*	8028	−1614	SNARE/SNAP receptor-related membrane protein/vesicle-mediated transport/intracellular protein trafficking/endosome–Golgi transport/membrane fusion/autophagy	FER, HOF, HOS
21	218987	0.007	5	29,090,184	*ZFYVE26*	44,865	−16,762	FYVE-type phosphatidylinositol 3-phosphate-binding protein involved in cytokinesis abscission, lysosome/autophagosome organization, and DNA double-strand break repair.	FER, HOF, HOS
22	430176	0.007	Z	79,188,522	*ALDH7A1*	18,188	42,551	Encodes an aldehyde dehydrogenase enzyme with NAD+-dependent oxidoreductase activity, involved in aldehyde metabolism/detoxification.	FER, HOF, HOS
23	430176	0.007	Z	79,188,522	*GRAMD3*	41,764	16,411	Encodes a membrane-associated GRAM domain-containing protein with transmembrane regions, potentially involved in protein binding and membrane-associated cellular processes.	FER, HOF, HOS
24	81215	0.007	2	9,917,342	*ZMYND11*	102,879	39,698	Nuclear chromatin reader involved in H3.3K36me3 recognition, transcriptional co-repression, RNA polymerase II elongation regulation, and chromatin organization.	FER, HOF, HOS
25	430522	0.007	Z	81,913,924	*LOC112530593*	12,606	−24,520	-	FER, HOF, HOS
26	430522	0.007	Z	81,913,924	*ZCCHC7*	104,325	28,326	Nuclear RNA-binding protein involved in TRAMP complex-associated RNA surveillance, RNA quality control (QC), and polyadenylation-dependent RNA degradation.	FER, HOF, HOS
27	199361	0.006	4	67,098,778	*GABRA2*	62,259	36,397	Encodes a ligand-gated chloride ion channel/receptor involved in chloride ion transport, GABAergic synaptic transmission, regulation of postsynaptic membrane potential, and inhibitory synapse assembly.	FER, HOF, HOS
28	199361	0.006	4	67,098,778	*LOC107053238*	29,948	−22,126	-	FER, HOF, HOS
29	91140	0.006	2	38,564,718	*EOMES*	4772	22,862	Transcription regulator/DNA-binding transcription factor/chromatin remodeling/developmental regulation	FER, HOF, HOS
30	42536	0.006	1	112,323,748	*FUNDC1*	16,551	−10,717	Encodes a mitochondrial outer membrane protein that acts as an activator of hypoxia-induced mitophagy and contributes to mitochondrial QC.	FER, HOF, HOS
31	42536	0.006	1	112,323,748	*KDM6A*	149,968	176,942	Nuclear JmjC-domain histone demethylase involved in H3K27me2/H3K27me3 demethylation, chromatin regulation, and gene expression control.	FER, HOF, HOS
32	42536	0.006	1	112,323,748	*LOC107055549*	13,642	20,433	-	FER, HOF, HOS
33	218597	0.006	5	28,319,218	*GALNT16*	60,479	70,369	Encodes a Golgi-associated glycosyltransferase that catalyzes the initial step of mucin-type O-linked glycosylation by transferring N-acetyl-D-galactosamine to serine or threonine residues on protein substrates.	FER, HOF, HOS
34	218976	0.006	5	29,065,498	*ARG2*	17,065	−9282	Arginase enzyme involved in L-arginine metabolism, nitrogen metabolism, and regulation of immune/inflammatory responses.	FER, HOF, HOS
35	218976	0.006	5	29,065,498	*PIGH*	6917	−1445	PIGH family membrane protein involved in GPI-anchor biosynthesis and post-translational modification of GPI-anchored proteins.	FER, HOF, HOS
36	91101	0.006	2	38,462,015	*SLC4A7*	133,258	78,180	Anion exchanger/sodium–bicarbonate transporter/intracellular pH regulation/ion homeostasis	FER, HOF, HOS
37	199558	0.006	4	67,570,591	*GNPDA2*	7654	−22,413	Cytoplasmic glucosamine-6-phosphate deaminase involved in carbohydrate and amino sugar metabolism.	FER, HOF, HOS
38	199558	0.006	4	67,570,591	*GUF1*	23,612	−23,596	Mitochondrial GTPase involved in mitochondrial ribosome binding, translation regulation, and mitochondrial protein synthesis.	FER, HOF, HOS
39	172509	0.006	3	107,651,719	*TRNAW-CCA*	72	−748	transfer RNA tryptophan	FER, HOF, HOS
40	199588	0.006	4	67,644,422	*YIPF7*	13,502	7427	YIP1 family multi-pass membrane protein involved in ER–Golgi vesicle transport, Golgi vesicle fusion, and intracellular membrane trafficking.	FER, HOF, HOS
41	270236	0.006	8	7,378,313	*LOC112532958*	133	17,109	-	FER, HOF, HOS
42	270236	0.006	8	7,378,313	*RFWD2*	128,131	127,649	COP1 family RING-type E3 ubiquitin ligase involved in protein ubiquitination and ubiquitin-dependent proteasomal protein degradation.	FER, HOF, HOS
43	270236	0.006	8	7,378,313	*TNR*	52,613	−8613	Secreted extracellular matrix glycoprotein involved in cell adhesion, extracellular matrix organization, cell migration, and nervous system development.	FER, HOF, HOS
44	91019	0.006	2	38,189,265	*LRRC3B*	44,358	67,434	Single-pass plasma membrane LRRC3 family protein potentially involved in protein binding and signaling receptor activity.	FER, HOF, HOS
45	91019	0.006	2	38,189,265	*NEK10*	211,881	22,472	NEK family serine/threonine protein kinase involved in protein phosphorylation, G2/M cell cycle regulation, and ERK1/2 signaling.	FER, HOF, HOS
46	263428	0.006	7	29,191,714	*DPP10*	359,956	15,688	Encodes a membrane-associated dipeptidyl peptidase/serine-type peptidase-like protein involved in proteolysis, potassium channel regulation, and potassium ion transmembrane transport.	FER, HOF, HOS
47	263428	0.006	7	29,191,714	*LOC112532778*	71,066	51,756	-	FER, HOF, HOS
48	172507	0.006	3	107,648,351	*PKHD1*	250,539	44,266	Membrane-associated glycoprotein involved in cilium assembly, centrosome regulation, calcium homeostasis, cell polarity, epithelial organization, and cell signaling.	FER, HOF, HOS
49	54002	0.006	1	141,213,374	*MIR1632*	91	24,523	-	FER, HOF, HOS
50	91145	0.006	2	38,581,424	*LOC101749416*	162,201	7996	-	FER, HOF, HOS
51	218792	0.006	5	28,703,531	*RAD51B*	364,933	122,312	RAD51 paralog/RecA-like DNA repair protein involved in homologous recombination, double-strand break repair, replication fork maintenance, and genome stability.	FER, HOF, HOS
52	362033	0.006	17	6,075,602	*FNBP1*	93,160	45,477	Encodes an FNBP1 family protein involved in endocytosis, signal transduction, lipid/protein binding, and membrane–cytoskeleton organization.	FER, HOF, HOS
53	30114	0.006	1	81,181,047	*NHLH2*	1301	−22,394	Nuclear bHLH DNA-binding transcription factor involved in transcriptional regulation, cell differentiation, nervous system development, and reproductive-related developmental processes.	FER, HOF, HOS
54	30114	0.006	1	81,181,047	*SLC22A15*	44,155	64,026	MFS/SLC-like membrane transporter involved in amino-acid betaine transport, organic cation transport, and small-molecule transmembrane transport.	FER, HOF, HOS
55	430379	0.006	Z	80,385,721	*LOC112530553*	635	35,540	-	FER, HOF, HOS
56	430379	0.006	Z	80,385,721	*LOC112530554*	1564	24,725	-	FER, HOF, HOS
57	153879	0.006	3	63,963,035	*NT5DC1*	120,970	35,073	5′-nucleotidase/deoxyribonucleotidase hydrolase involved in nucleotide metabolism and metal ion-dependent catalytic activity.	FER, HOF, HOS
58	429106	0.006	Z	67,707,887	*ELAVL2*	85,034	84,305	RNA-binding protein with RRM domains, potentially involved in the post-transcriptional regulation of gene expression.	FER, HOF, HOS
59	91278	0.006	2	39,004,490	*RBMS3*	698,816	151,192	RRM domain-containing RNA-binding protein involved in mRNA 3′-UTR binding, post-transcriptional gene regulation, and translation control.	FER, HOF, HOS
60	146795	0.006	3	47,479,346	*SASH1*	530,243	139,469	Scaffold/adaptor protein involved in TLR4–NF-κB signaling, K63-linked ubiquitination, p38 MAPK activation, inflammation, cell migration, and angiogenesis	FER, HOF, HOS
61	172445	0.006	3	107,525,196	*EFHC1*	17,890	21,674	Cytoskeleton- and cilium-associated protein involved in alpha-tubulin binding, microtubule organization, cell division, and ciliary motility.	HOF, HOS
62	172445	0.006	3	107,525,196	*IL17F*	5036	−31,057	Encodes a secreted cytokine belonging to the IL-17 family, involved in inflammatory response, cytokine activity, and cytokine–cytokine receptor interaction.	HOF, HOS
63	172445	0.006	3	107,525,196	*MCM3*	9459	−18,370	MCM family DNA helicase involved in DNA replication initiation, replication fork progression, and cell cycle regulation.	HOF, HOS
64	172445	0.006	3	107,525,196	*PAQR8*	14,515	235	ADIPOR family multi-pass membrane receptor-like protein involved in steroid hormone response and membrane-associated signaling.	HOF, HOS
65	172445	0.006	3	107,525,196	*TRAM2*	15,736	38,475	Multi-pass membrane protein involved in protein translocation, ER membrane protein insertion, and ceramide biosynthesis/sphingolipid metabolism.	HOF, HOS

FER = fertility rate, HOF = hatchability of fertile eggs, HOS = hatchability of eggs set.

## Data Availability

The original contributions presented in the study are included in the article; further inquiries can be directed to the corresponding author.
